# *Angiostrongylus vasorum* in Romania: an extensive survey in red foxes, *Vulpes vulpes*

**DOI:** 10.1186/s13071-017-2270-x

**Published:** 2017-07-12

**Authors:** Georgiana Deak, Călin M. Gherman, Angela M. Ionică, Alexandru D. Vezendan, Gianluca D’Amico, Ioana A. Matei, Aikaterini A. Daskalaki, Ionuț Marian, Aurel Damian, Vasile Cozma, Andrei D. Mihalca

**Affiliations:** 1 Department of Parasitology and Parasitic Diseases, University of Agricultural Sciences and Veterinary Medicine, Cluj-Napoca, Romania; 2 Department of Comparative Anatomy, University of Agricultural Sciences and Veterinary Medicine, Cluj-Napoca, Romania

**Keywords:** *Angiostrongylus vasorum*, Romania, Red fox, *Vulpes vulpes*

## Abstract

**Background:**

*Angiostrongylus vasorum* is the causative agent of canine angiostrongylosis, a severe snail-borne disease of dogs. Red foxes are important natural reservoirs of infection, and surveys of foxes provide a more objective picture of the parasite distribution. Our aim was to investigate the possibility of the presence of *A. vasorum* in red foxes from the western part of Romania and to analyse the risk factors related to the sex, age and geographic origin of the foxes. Between July 2016 and April 2017, 567 hunted red foxes from 10 counties of western Romania were examined by necropsy for the presence of lungworms.

**Results:**

Overall, the infection with *A. vasorum* has been found in 24 red foxes (4.2%) originating in four counties (Mureș, Hunedoara, Sălaj and Cluj). There was no significant difference between the prevalence in males and females, between juveniles and adults and between counties.

**Conclusions:**

This is the first report of autochthonous infections of *A. vasorum* in Romania, showing a relatively low prevalence and extending eastwards the known distributional range of this parasite in Europe. The presence of autochthonous cases in domestic dogs in Romania remains to be confirmed by further studies.

## Background

*Angiostrongylus vasorum*, or the French heartworm, is the causative agent of canine angiostrongylosis, a severe snail-borne disease of dogs, with an almost worldwide distribution (Europe, South America, North America and Africa) [1]. Since its description in France [2], the parasite has been found in several European countries [3], being nowadays considered an emerging parasite [4]. Despite its wide geographical distribution, the presence of *A. vasorum* throughout its range seems to be patchy, with endemic disease foci surrounded by areas with sporadic cases [3]. Although during the last years the research on this parasite has intensified, the actual distribution is considered unknown [5], mainly because of unreported cases and limited awareness of clinicians [4]. In the last two decades, the presence of *A. vasorum* has been reported for the first time in several European countries (Table 1).

**Table 1:** Year of the first country report of autochthonous cases of *Angiostrongylus vasorum* in Europe in the last two decades

Year of the first report of autochthonous cases^a^	Country	Host species	Method	Reference
2002	Croatia	Red fox	Necropsy	[39]
2003	Germany	Domestic dog	Baermann	[25]
2003	Hungary	Red fox	Necropsy	[40]
2003	Sweden	Domestic dog	Necropsy	[41]
2004	Iceland	Domestic dog	Baermann	[42]
2007	Greece	Domestic dog	Sedimentation	[43]
2008	Netherlands	Domestic dog	Baermann	[44]
2013	Poland	Domestic dog	ELISA	[45]
2013	Slovakia	Domestic dog	Baermann	[46]
2014	Serbia	Domestic dog	Baermann	[47]
2014	Czech Republic	Domestic dog	Baermann	[48]
2015	Belgium	Domestic dog	Necropsy, PCR	[49]
2015	Albania	Domestic dog	Baermann	[50]
2017	Romania	Red fox	Necropsy	present study

^a^Only the countries where the first report of *A. vasorum* was published in the last 20 years are included. Countries where the parasite was reported before, are not included. This is to highlight the increased interest and/or possible emergence of *A. vasorum*

Red foxes are known to be important natural reservoirs of parasitic infection for domestic animals and humans across their distribution range [6]. In Romania, red foxes have been demonstrated as carriers of a wide range of parasites: *Trichinella* spp. [7], *Echinococcus multilocularis* [8], ticks and tick-borne bacteria [9–12], *Toxoplasma gondii* and *Neospora caninum* [13], *Eucoleus aerophilus* [14] and *Hepatozoon canis* [15].

The spatial model suggested by Morgan et al. [5] includes the western part of Romania as a risk area for the presence of *A. vasorum*, but so far there are no confirmed autochthonous cases. Our aim was to investigate the possibility of the presence of *A. vasorum* in red foxes from the western part of Romania and to analyse the risk factors related to the sex, age and geographic origin of the foxes.

## Methods

### Samples

Between July 2016 and April 2017, 567 red foxes, were collected by hunters in 10 counties (Table 2) (through the County Veterinary Authority) of Romania. For safety reasons, only foxes which were confirmed as negative for rabies were examined. Prior to necropsy, all foxes have been deep frozen. During the necropsy, the right heart and pulmonary arteries were opened and carefully checked for the presence of parasites. All nematodes were collected in 70% ethanol and morphologically identified [16]. For each fox, the location, sex and age (young, less than one-year-old; and adult, more than one-year-old, according to Harris [17]) was noted.

**Table 2:** Presence of *Angiostrongylus vasorum* in red foxes, *Vulpes vulpes*, from western Romania

County	Total		Males		Females		Adults		Young	
	*n*	+	*n*	+	*n*	+	*n*	+	*n*	+
Arad	30	0	14	0	16	0	11	0	19	0
Bihor	41	0	28	0	13	0	38	0	3	0
Caraș-Severin	18	0	9	0	9	0	12	0	6	0
Cluj	33	1 (3.0%)	16	0	17	1 (5.9%)	24	1 (4.2%)	9	0
Gorj	99	0	62	0	37	0	70	0	29	0
Hunedoara	61	5 (8.2%)	30	5 (16.7%)	31	0	45	3 (6.7%)	16	2 (12.5%)
Maramureș	23	0	15	0	8	0	12	0	11	0
Mureș	156	17 (10.9%)	81	8 (9.9%)	75	9 (12.0%)	108	8 (7.4%)	48	9 (18.8%)
Sălaj	25	1 (4.0%)	16	1 (6.3%)	9	0	12	1 (8.3%)	13	0
Satu-Mare	82	0	52	0	30	0	47	0	35	0
Total	567	24 (4.2%)	322	14 (4.3%)	245	10 (4.1%)	378	13 (3.4%)	189	11 (5.8%)

### Statistical analysis

Statistical analyses were performed using EpiInfo™ 7 software (CDC, USA). The mean intensity and prevalence of infection and its 95% confidence interval (95% CI) were calculated. The differences among positive groups were assessed by means of chi-square testing and were considered significant if *P-*values were lower than 0.05.

### Molecular identification

Genomic DNA was extracted from 10 adult females using a commercial kit (Isolate II Genomic DNA Kit, Bioline, London, UK) according to the manufacturer’s instructions. For each nematode, PCR amplifications of a partial mitochondrial cytochrome *c* oxidase subunit 1 (*cox*1, ∼700 bp) gene and the internal transcribed spacer 2 (ITS2, ∼500 bp) of the rRNA gene, were performed according to literature [18, 19]. Amplicons were purified using a commercial kit (Isolate II PCR and Gel Kit, Bioline, London, UK) and sequenced (performed by Macrogen Europe, Amsterdam). The newly generated sequences were compared to those available in the GenBank by Basic Local Alignment Search Tool (BLAST) analysis.

## Results

**Fig. 1:**
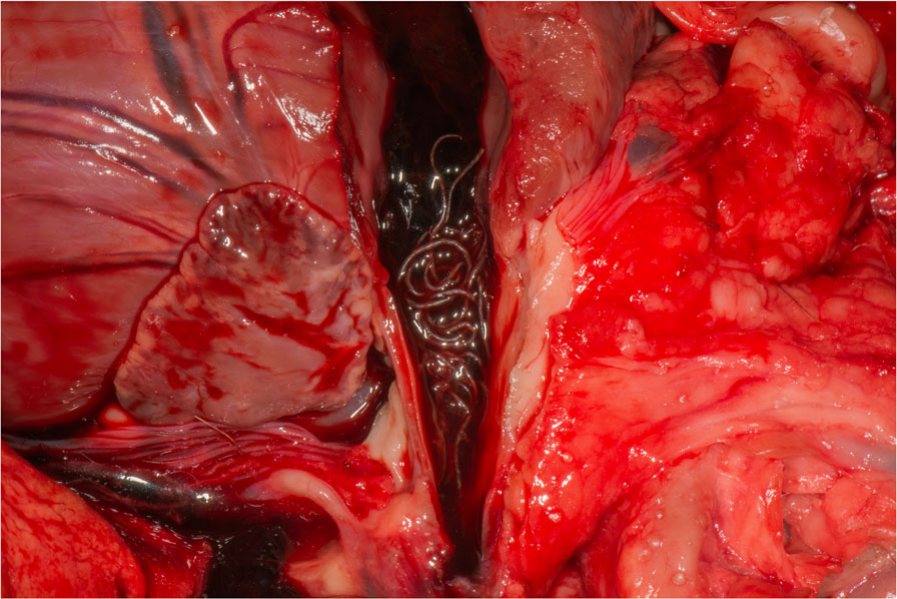
*Angiostrongylus vasorum* in the pulmonary artery of a red fox, *Vulpes vulpes* in Romania

All nematodes collected form the pulmonary arteries and right ventricle (Fig. 1) of foxes were identified based on morphological criteria as *A. vasorum*. Ten nematodes were randomly selected for further molecular confirmation. BLAST analysis revealed a 100% identity to other *A. vasorum* sequences (GQ982791, GQ982741 for *cox*1; GU045374, EU627596, EU915248 for ITS2).

**Fig. 2:**
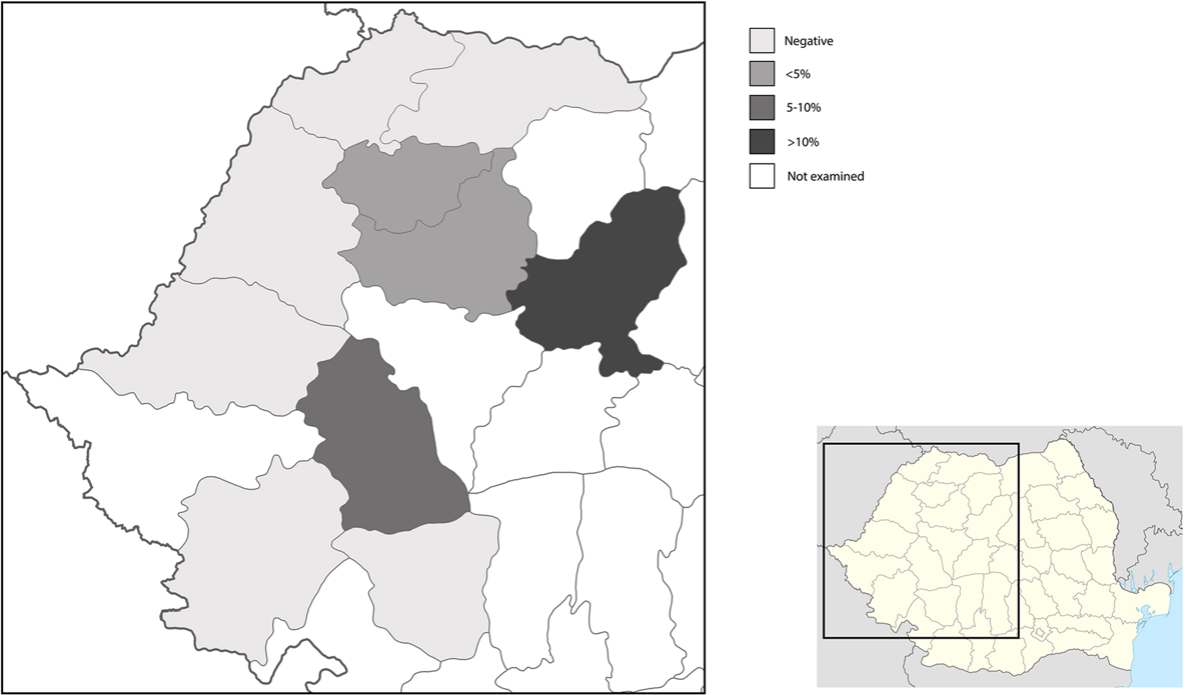
Prevalence map of *A. vasorum* in red foxes in western Romania

Out of the 567 red foxes examined, 24 (4.2%; 95% CI: 2.86–6.22) were positive for *A. vasorum* infection (Table 2). *Angiostrongylus vasorum* was found in four counties (Fig. 2), with a prevalence ranging between 3.0 (95% CI: 0.08–15.76) and 10.9% (95% CI: 6.52–16.98). There was no significant difference between the prevalence in males and females (*χ* ^2^ = 0, *df* = 1, *P* = 1), between juveniles and adults (*χ* ^2^ = 1.22, *df* = 1, *P* = 0.26) and between counties (*χ* ^2^ = 3.03, *df* = 3, *P* = 0.38).

The intensity of infection varied between 1 and 57 nematodes per positive animal (mean intensity 11.8). The mean intensity in adult foxes was 10.6 and in juveniles 14.7. The mean intensity in female foxes was 10.0 in males and 11.5 in females. The average sex ratio (M:F) in the parasite infrapopulation was 0.32 (range 0.8–1.5).

## Discussion

In general, foxes are considered to be important reservoirs of infection with *A. vasorum* for domestic dogs [20, 21]. A study in Canada showed that the infection in foxes has established long before the first canine cases were recorded [22]. Most studies indicate that, in general, the local prevalence in foxes is higher compared to dogs [23]. Furthermore, Helm et al. [4] suggested that surveys of foxes provide a more objective picture of the parasite distribution. Although foxes are essential in the maintenance of infection foci, dogs are considered to have the main role in the geographical spreading of the parasite, mainly due to the more intense movements (i.e. importation, tourism) [4]. Interestingly, despite existing parasitological surveys in foxes, most of the first country reports from the last two decades in Europe, originate in domestic dogs (Table 1). The only exceptions are countries form the margin of the distribution area of *A. vasorum* (Croatia, Hungary, Romania), where foxes were found infected before dogs (Table 1).

The recorded prevalence of infection with *A. vasorum* in red foxes in Europe is between 5.0 and 78.2% [4, 24] but there is a high variation across different regions. The average prevalence in our study was 4.2%, at the lower limit of the overall range in Europe. This is somehow expected, as Romania is at the eastern margin of the distributional range of *A. vasorum*. Similar prevalence rates were recorded in foxes in Hungary (5.0%) [25], Poland (5.2–5.3%) [26] and Portugal (7.1%) [27]. However, in foxes from the endemic areas of Europe the prevalence is generally higher: UK (18.3%) [28], Spain (33.3%) [29], Ireland (49.3%) [30], Denmark (48.6%) [31] and Italy (78.2%) [24].

In our study, young foxes (less than one-year-old) were more commonly infected with *A. vasorum* than adults, but with no statistical significant differences. However, several studies from highly endemic areas have shown an opposite trend, with higher prevalence in adult foxes [31]. In other studies from less endemic areas, there was no significant difference between the prevalence in adult and young foxes [32]. In an experimental infection study with *A. vasorum* in red foxes, Webster et al. [33] proved that adult animals are more resistant than juveniles. The higher prevalence in young animals has been documented on several occasions also in dogs (as reviewed by Helm et al. [4]). Several hypotheses have been suggested to explain this pattern, such as the inquisitive nature of young animals making them more likely to be exposed to snails [34], age-related differences in dietary and scavenging behaviour [4] or an increased acquired immunity with age [33]. There is no consistent opinion on the gender predisposition neither in dogs, nor in foxes, and most reports failed to find increased rates of infection in males or females [4].

Interestingly, from the 10 examined counties, the infection with *A. vasorum* in foxes has been found only in four. The counties located at the western border of Romania (i.e. Satu-Mare, Bihor, Arad), despite a relatively high number of foxes examined, were all negative. All these counties have a predominantly lowland elevation (< 130 m above sea level, masl). The infection was present only in counties with predominant altitudes between 400 and 600 masl. The only exception was Gorj County (predominantly hilly), where no cases were found despite the high number of foxes examined. The absence of *A. vasorum* in Maramureș and Caraș-Severin counties might be related also to the low number of examined samples. Previously, larval stages resembling *A. vasorum* have been found in dogs from the western part of Romania (Timiș County). However, no details on the parasite identification, molecular identity or travel history of the dogs have been provided [35] so the autochthonous nature of these cases remains to be confirmed. Recently, two other species of the genus have been documented in Romania: *A. chabaudi* in wildcats [36] and *A. daskalovi* in badgers [37]. Recently, a large-scale serological study in red foxes from Switzerland suggested that foxes may have an increased parasite tolerance, allowing the long-term survival of *A. vasorum* in these canids. This might explain the importance of red foxes in the epidemiology of *A. vasorum* across Europe [38].

Although this is the first report of autochthonous *A. vasorum* infection in Romania, the absence of this parasite so far is probably related to a poor surveillance of wild canids and a lack of awareness among small animal clinicians rather than representing a situation of an emerging disease. The clinical signs in domestic dogs are characteristic, consisting most commonly in respiratory signs (coughing, dyspnoea, tachypnea, gagging) and coagulopathies (haemorrhagic diatheses) [4]. However, they are not pathognomonic, and a confirmatory test (usually larvoscopy or serology) is needed [4]. It is known that client and veterinarian awareness on canine angiostrongylosis is poor in non-endemic areas [4] and this might result in significant underdiagnosis. This is why, the confirmation of *A. vasorum* in foxes in Romania, opens new differential diagnostic opportunities in the canine medicine.

## Conclusions

This is the first report of autochthonous *A. vasorum* infection in Romania, showing a relatively low prevalence and extending eastwards the known distributional range of this parasites in Europe. The presence of autochthonous cases in domestic dogs in Romania remains to be confirmed by further studies.
